# Motion: enhancing signals and concealing cues

**DOI:** 10.1242/bio.058762

**Published:** 2021-08-20

**Authors:** Eunice J. Tan, Mark A. Elgar

**Affiliations:** 1 Division of Science, Yale-NUS College, Singapore 138527, Singapore; 2 School of BioSciences, University of Melbourne, Melbourne, Victoria 3010, Australia

**Keywords:** Motion, Visual signal, Camouflage, Animal movement, Colour pattern

## Abstract

Animal colour patterns remain a lively focus of evolutionary and behavioural ecology, despite the considerable conceptual and technical developments over the last four decades. Nevertheless, our current understanding of the function and efficacy of animal colour patterns remains largely shaped by a focus on stationary animals, typically in a static background. Yet, this rarely reflects the natural world: most animals are mobile in their search for food and mates, and their surrounding environment is usually dynamic. Thus, visual signalling involves not only animal colour patterns, but also the patterns of animal motion and behaviour, often in the context of a potentially dynamic background. While motion can reveal information about the signaller by attracting attention or revealing signaller attributes, motion can also be a means of concealing cues, by reducing the likelihood of detection (motion camouflage, motion masquerade and flicker-fusion effect) or the likelihood of capture following detection (motion dazzle and confusion effect). The interaction between the colour patterns of the animal and its local environment is further affected by the behaviour of the individual. Our review details how motion is intricately linked to signalling and suggests some avenues for future research.

This Review has an associated Future Leader to Watch interview with the first author.

## Abstract (precis)

**Summary:** While motion can reveal information about the signaller, motion can also be a means of concealing cues by reducing the likelihood of detection or the likelihood of capture following detection.

## Introduction

Historically, investigations of the evolutionary significance of colour and colour patterns in nature have assumed, either implicitly or explicitly that the organism is static. Early accounts of the significance of bright colouration, camouflage or mimicry relied on images, historically through drawings, paintings and more recently through photography. Thus, Bates' (1861) account of the remarkable mimicry in tropical butterflies is clearly apparent when the illustrations of models and mimics are placed side by side. However, all animals, with the exception of those permanently attached to the substrate (e.g. limpets), move and this is likely to have an impact on the functional significance of their colour patterns. Remaining stationary is, in fact, an unusual and specific behaviour: the evolutionary significance of thanatosis, where animals abruptly cease moving and adopt a posture that mimics death (e.g. Ruxton et al., 2018) was noted by Charles Darwin, who suggested an anti-predator function (Romanes and Darwin, 1885). For example, beetles that detect a disturbance to the leaf or branch where they are resting, will drop off the substrate and fall to the ground where they remain immobile for some time (Crowson, 1981). The critical assumption of the anti-predator function of thanatosis is that immobile individuals are less conspicuous than moving individuals (Skelhorn, 2018). In other words, a lack of movement in animals is unusual, and thus investigations of static colour and colour patterns may be incomplete.

While animal movement can draw unwanted attention, it is also an effective means of conveying animal signals. This may be a means of alerting the receiver to an imminent, information-rich signal or a subsequent action (e.g. Peters et al., 2003; Számadó, 2015). Head-bobbing in *Anolis* lizards apparently functions to draw attention to the individual, thereby ensuring that elements of the subsequent display are not missed (Ord and Stamps, 2008), while the duration of an introductory ‘tail-flick’ in Jacky dragons affects the efficacy of the subsequent visual display (Peters and Evans, 2003). Movement may also enhance, amplify or reveal a feature of the animal: shimmering reveals the spectacular colour patterns of a peacock's train (e.g. Petrie et al., 1991), while the colour patterns of the male peacock spider are revealed only when the abdomen flap is raised (e.g. Girard et al., 2011). Animal movement may form part of a defence strategy by concealing the individual against the moving background (Bian et al., 2016), or by confusing or distracting an imminently attacking predator: the broken-wing behaviour of ground-nesting birds may lure enemies away from the nest (Deane, 1944), while an unexpected and sudden deimatic display of conspicuous colours may act as a deterrent (e.g. Olofsson et al., 2012 and Umeton et al., 2019). Finally, the patterns of movement can convey information. The honeybee ‘dance’ is arguably one of the first descriptions of animal movement as a source of information: returning foraging and scouting workers use specific movements to convey information to their nestmates about the nature and location of the food and other resources (von Frisch, 1967; Seeley, 2003).

**Fig. 1.**
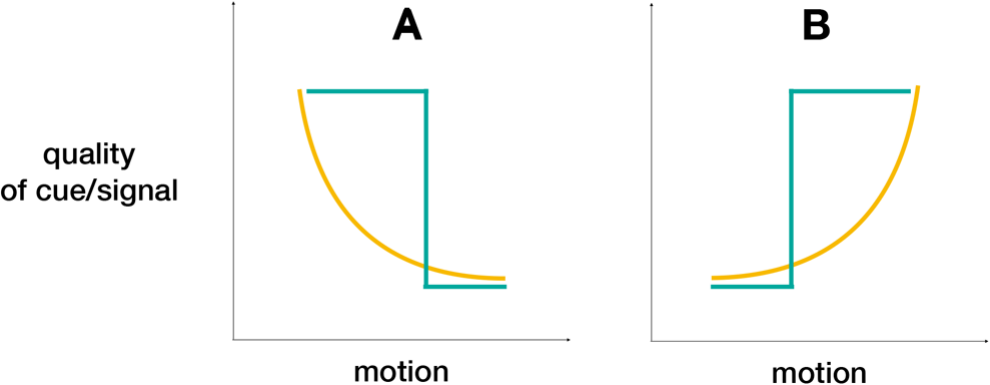
Effect of motion on the capacity of receivers to detect a signal or cue as a continuous or binary (or step) function. (A) Motion leads to decreasing quality of the signal/cue. (B) Motion leads to increasing quality of the signal/cue. Turquoise line indicates a binary function of signal/cue to motion, yellow line indicates a continuous function of signal/cue to motion.

This raises the broader question of whether the ability of receivers to detect the information conveyed by a signal or cue is a continuous or binary (i.e. step) function of animal movement (Fig. 1). In other words, is any movement sufficient to prevent the signal from conveying the information, or does the strength of the signal, and thus the quality of the information, attenuate linearly (or non-linearly) with movement? In some contexts, the presence or absence of any movement may be sufficient to alter the receiver's capacity to detect the signal or cue (or its source). However, in other contexts the nature of the information may crucially vary with quantitative differences in movement. For instance, is the act of swaying in phasmids (discussed further in Section 4.1) at any speed sufficient to provide protection, or does the speed of swaying have to be aligned with that of the moving background vegetation? The speed of movement could make the signal impossible to discern, or the movement of the colour pattern could result in a diminished message. In both instances, the threshold will depend on the visual acuity of the receiver and may be a current source of unexplained variation in mate choice or other contexts. Courtship in the peacock spider *Maratus volans* involves a combination of waving the third legs, shimmering of the opisthosomal (abdominal) fan, and moving the entire body from side to side (Girard et al., 2011). The tiny size of these spiders may allow them to create such a remarkable and highly conspicuous display without attracting predators, but the question remains: to what extent does the different degree of movement conceal or enhance the signal to the intended female receivers? Even though peacock spiders can and do move their abdomen, the ‘fan dance’ also involves protracted periods of motionless display of the opisthosomal fan, which may allow the receiver to fully discern the information conveyed by the courting male. In some contexts, the presence or absence of movement may be sufficient to alter the receiver's capacity to detect the signal or cue, but in other contexts, the nature of the information may vary crucially with quantitative differences in movement.

### Motion, signals and cues

It is important to distinguish between signals and cues (Maynard Smith and Harper, 2003; Stevens, 2013). Animals that move are likely to increase their conspicuousness: indeed, a mere breath can reveal their presence to prey and predators (Tomita et al., 2018). These movements may either (1) act as cues, because they provide information about the individual, including their location, but the movement has not evolved for that purpose; or (2) form an integral part of a signal, which has evolved precisely to influence the behaviour of the viewing receiver and typically benefits both signaller and receiver. The movement of animals can simultaneously generate signals and cues. For example, the highly conspicuous courtship behaviour of male manakins involves a great deal of movement (Chapman, 1935), and this display likely provides females with information about the male. At the same time, such a highly conspicuous activity can also act as a cue for unintended receivers, or eavesdroppers. Thus, the traditional areas where male manakins display are typically located in the forest shade, where the males are less conspicuous against the background when viewed from a distance by potential predators (Heindl and Winkler, 2003).

The methods of investigating the evolutionary significance of visual signals and cues have become increasingly sophisticated, and now incorporate the role of light (e.g. Endler, 1993; 2012) and background (e.g. Endler, 1990; Tan et al., 2017), and use digital photography and modelling (van den Berg et al., 2020) to take into account the visual acuity of the intended and unintended receivers (e.g. Schnaitmann et al., 2020 and Silvasti et al., 2021; also see review by Osorio and Vorobyev, 2008). Nevertheless, while technically more sophisticated, these investigations remain largely shaped by a focus on stationary animals in a static heterogeneous background (Cuthill, 2019; Cuthill et al., 2017; Hughes et al., 2019). For instance, static prey items are used to understand the effectiveness of countershading (Cuthill et al., 2016; Penacchio et al., 2017), predator’ responses to prey contrast against complex backgrounds (Dimitrova and Merilaita, 2010) and the effect of background complexity on detection distance (Xiao and Cuthill, 2016). Indeed, arguably the most convincing evidence of the effectiveness of camouflage in nature comes from field experiments involving eggs, which remain stationary in the nest (e.g. Troscianko et al., 2016; Tinbergen et al., 1962; also see recent reviews on camouflage strategies Galloway et al., 2020; Cuthill, 2019, Merilaita et al., 2017). Using static model prey may be appropriate in systems with slow-moving organisms, because the interaction of movement and colour patterns may be negligible. For example, relatively slow-moving leaf beetles can be much smaller than their background leaves (Tan et al., 2017) and thus the background does not change rapidly as they move. However, our understanding of larger, more mobile organisms is less well-served by experimental studies of stationary individuals, since their natural background will change more rapidly as they move. Such animal mobility may create a highly variable background if it comprises heterogeneous vegetation or substrate. Moreover, this variability is further increased if the background also moves, which is referred to here as a dynamic background (Cuthill et al., 2019).

### Review outline

**Fig. 2.**
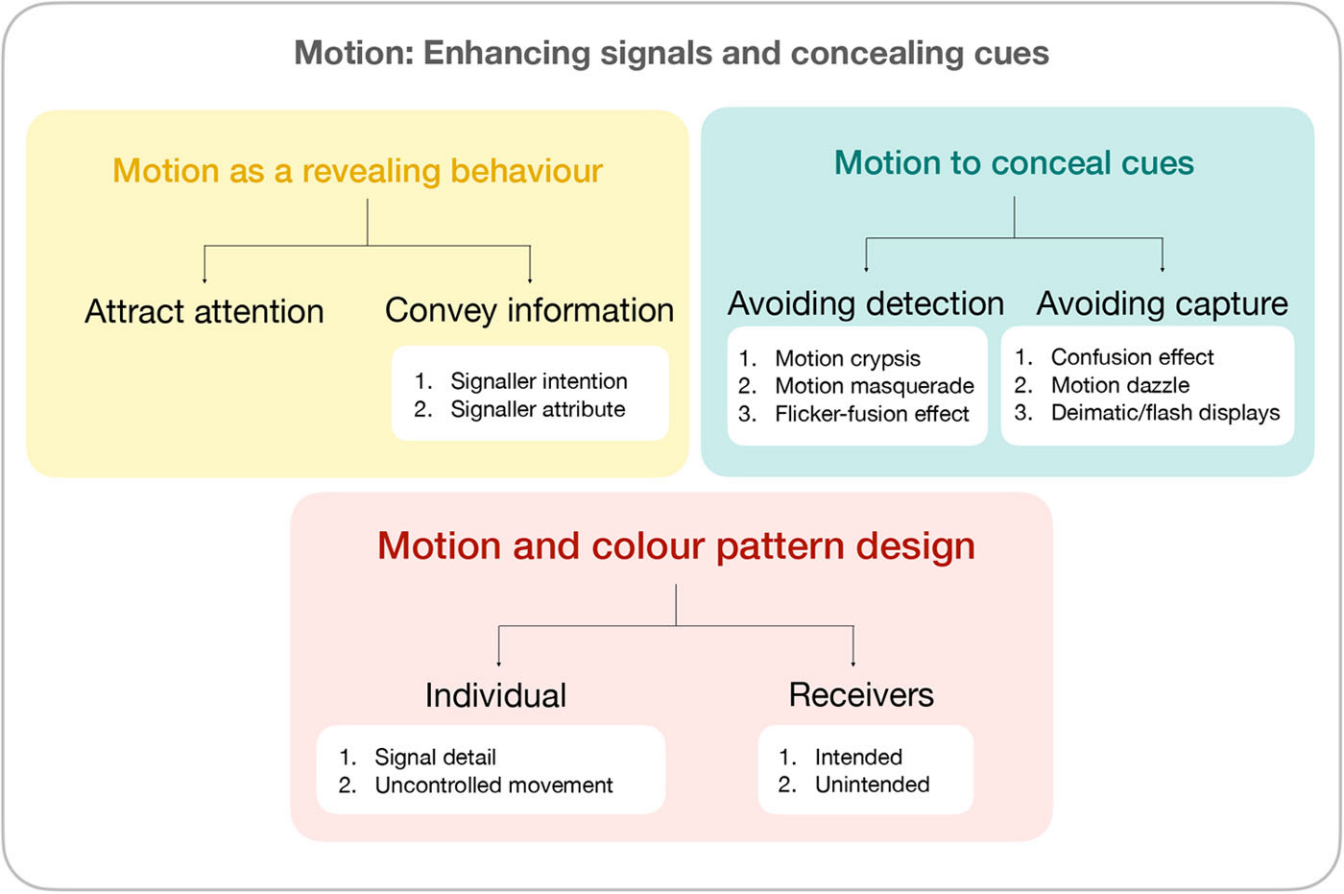
**Schematic overview of article.** Schematic overview of this Review, reflecting the organisation of the article and the main concepts discussed.

Our review emphasises that investigations of the evolutionary significance and function of visual cues and signals should pay attention to the movement of their source, and in particular how the movement of animals can enhance or conceal both signals and cues (see Fig. 2). Drawing on a conventional framework of signalling theory (e.g. Maynard Smith and Harper, 2003), we discuss how motion is linked to signalling, both by drawing attention to the signaller and by providing information about the signaller. Next, we discuss motion as a means of concealing cues, by reducing the likelihood of detection or the likelihood of capture following detection. Finally, we explore how animal colour patterns can be shaped by an interaction between the behaviour of the individual and its local habitat. Key terms referred to in this review are listed in Box 1.

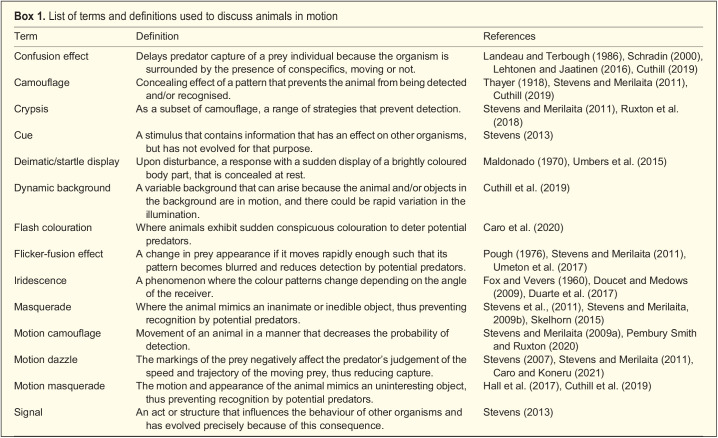

## Motion as revealing behaviour

Animal motion, either as a consequence of moving from one place to another or of particular behaviours, can provide a locational cue for natural enemies. For instance, motionless, cryptically patterned stickleback larvae that matched their background were less likely to be detected and attacked, compared with more active individuals (Ioannou and Krause, 2009). Thus, unsurprisingly, animal motion can act as a signal or can amplify the signal (*sensu* Hasson and Hamilton, 1989), thereby revealing information about the intention or quality of the signaller.

### Motion to attract attention

Visual signals transfer information only when they are detected by the receiver, which means that the signaller must be in the line of sight of the receiver. For species that transmit signals across long distances, it may be necessary to draw the attention of the intended receiver prior to projecting the signal, especially if the signal is complex and the information is intrinsically linked to that complexity. Accordingly, signals may be preceded by an ‘alert’ component, a pattern that is common in acoustically-signalling species (Számadó, 2015) but has received less attention in visually-signalling species (Ord and Stamps, 2008). For example, territorial species of *Anolis* lizards advertise territorial boundaries with dynamic visual displays that include head-bobbing and repeated extensions of the colourful dewlap. In some species, individuals make exaggerated ‘introductory’ movements that increase the likelihood of signal detection (Ord and Stamps, 2008). In other words, the movement of the animal acts to amplify the alert signals, an action that is likely to be especially important when the dynamic visual displays take place against a dynamic background (Fleishman, 1992). Indeed, Jacky lizards spend more time tail-flicking, an alert movement that occurs before the territorial display, on more windy days with greater movement of the background vegetation (Peters et al., 2007).

### Motion that conveys information about the signaller

#### Motion indicating intention

Motion can indicate the possession of defences, without actually executing them. For example, some chrysomelid beetles that perceive a threat will evert glands that contain chemical defences (Pasteels et al., 1982) and tawny crazy ants adopt a ritualised aggressive behaviour, where they curl their abdomen at their opponents but refrain from spraying formic acid (LeBrun et al., 2019). Presumably, this threatening behaviour achieves a similar outcome but without incurring the costs of synthesising the chemical defences. Nevertheless, there is a degree of ambiguity whether these behaviours represent a signal, allowing the threatening receiver to withdraw, or simply represent a change in posture that is necessary for the animal to defend itself. The latter can be used by the antagonist as a cue indicating an imminently dangerous encounter. This ambiguity is nicely illustrated by spitting cobras, which tend to elevate the front body in a characteristic manner prior to spitting. Although this posture does not always indicate that the individual will spit (Rasmussen et al., 1995), it may discourage any further engagement by the potentially threatening antagonist. Distinguishing whether this movement represents a threatening signal, or a cue associated with an imminent ‘spit’ is likely to be challenging, but such movements seem more likely to represent highly reliable signals of intent for non-specialist, mildly threatening receivers. On the other hand, it could be a less reliable or less effective cue when the snake is confronted by specialist snake-eating predators, such as mongoose, that could modify their attack based on these cues. In other words, motion can represent a signal of intent, but the reliability of this signal can vary depending on the attributes of the receiver.

#### Motion indicating signaller attributes

Animals signal information about their attributes or qualities in different sensory modalities and contexts, such as mate choice, resource defence and anti-predator defence. These signals may be relatively simple and constrained to a single sensory modality, such as a sound or colour pattern, or a more complex synergy of different sounds, odours and colours that is often referred to as multimodal signalling (e.g. Higham and Hebets, 2013). For instance, peacock spiders use seismic and visual signals in sexual signalling (Girard et al., 2011), with certain colours particularly influencing female choice (Girard et al., 2018). Movement often forms a vital component of these multimodal signals, but it is not always clear whether receivers pay attention to the movement *per se* or to its consequence: for example, orthopterans communicate with conspecifics by stridulating specially modified body parts (e.g. Haskell, 1958, Eiriksson, 1993). Here, the information is conveyed by the sound rather than the movement, as the signaller is typically not within sight. In contrast, courting cursorial spiders create seismic signals by drumming with their leg and body parts, and receivers may pay attention to both the vibrations and the movement of those body parts responsible for the seismic signals (e.g. Elias et al., 2005 and Uetz et al., 2009). Indeed, adding movement to visual signals also allows individuals to provide a more contemporary account of their attributes than may be possible with colour patterns alone. This may be important to the receiver if those attributes change with time, and the colour patterns reflect an historical account of quality when they were generated. A contemporary link between the signal and the quality of the signaller is inherent in auditory, vibrational and olfactory modalities, and perhaps combining colour pattern and movement, if the latter is physiologically costly, provides a similarly contemporary signal of quality in visual systems.

The nature of movement, in terms of speed and direction, may be integral to the signal, as is the case for honeybee workers, whose ‘dance’ conveys information to their nestmates about the location and quality of food resources (von Frisch, 1967; Seeley, 1996). Male fiddler crabs wave their enlarged claws during social interactions, and the complexity of the waving movement provides specific information, with more complex waving associated with broadcasting male location, and simpler but more frequent waving associated with mate choice (Perez and Backwell, 2020). Movement can form an important feature of the agonistic behaviour of contesting conspecifics, providing information about the protagonists' combative or fighting ability (Hardy and Briffa, 2013). For example, contesting male ungulates engage in ritualised parallel walks, and although the function of this behaviour and the nature of the information being conveyed is unclear (Jennings et al., 2003), it seems unlikely that this information could be conveyed as effectively if the contestants remained stationary. On the other hand, movement can add ambiguity to the signal. Neighbouring colonies of meat ants *Iridomyrmex purpureus* deploy numerous workers to traditional display areas, located midway between neighbouring nests, where pairs of non-nestmate workers rapidly antennate each other (Van Wilgenburg et al., 2005). This collective signalling provides information about relative colony size – pairs of displaying workers may be interrupted by a third worker, whose colony identity typically reflects their relative abundance and thus signals relative colony size (Orbell et al., 2020). However, this signal may be vulnerable to deception, at least in the short term, because workers from one colony could artificially exaggerate their relative colony size by moving more rapidly and thereby interrupting displaying pairs more frequently (Orbell et al., 2020).

Animals may combine visual signals with movement, although it is not always clear whether the information is conveyed by the movement, the colour pattern, or both. For example, prey may alert predators that they have been detected, thereby discouraging the predator from continuing their hunt, since the likelihood of success through the element of surprise has been reduced. Stotting in ungulates, where all four legs of the prey animal are held stiff and off the ground, is a classic example of such signals to potential predators (Caro, 1986). In this case, a particular feature of the animal, such as conspicuous rump marks, may act as an amplifying agent that allows a more accurate assessment of the rate of stotting, which is the real display of quality (FitzGibbon and Fanshawe, 1988). Interestingly, the strength of this signal is correlated with the degree of concealing environment, with individuals stotting more frequently with increased vegetation height (Stankowich and Coss, 2007). Movement of a particular body part may also act to amplify the signal. For example, tail flashing in juncos is a courtship signal, but during winter may also act as a pursuit-deterrent signal to predators: field experiments revealed that the rate of tail flashing was higher in the presence of a model predator, but that individuals nevertheless reduced the rate of tail flashing with greater distance from the safety of cover (Ramesh and Lima, 2019). Here, the rate of tail flashing, rather than the colour characteristics of the tail, seems likely to be of most interest to the receiver.

While movement can clearly enhance the visual signal, for example in the spectacular courtship displays of many species of birds, such as lyrebirds (Dalziell et al., 2013), it can be a crucial factor maximising the intensity of iridescent signals (White et al., 2015), where the colour hue changes according to the viewing and/or illumination geometry (Stuart-Fox et al., 2021). The iridescent colour patterns on the train of a male peacock are especially evident when he shimmers or rattles the tail (Dakin and Montgomerie, 2013), and iridescent colour patterns are common in bird species that are active in bright environments (Seymour and Dean, 2010). When movement is crucial for conveying the signal, especially if it provides information about the quality of the signaller, the consequence is not necessarily the same. For example, male *Selasphorus platycercus* (broad-tailed hummingbirds) precisely coordinate their aerial dive display to ensure that the female witnesses a dramatic red flash as the colours abruptly change on their iridescent gorget (Hogan and Stoddard, 2018). In contrast, the flight path of *Calypte costae* (Costa's hummingbird) and *Calypte anna* (Anna's hummingbird) ensures that the gorget retains a constant hue (Simpson and McGraw, 2019). Depending on the context, movement can be a crucial factor affecting the intensity and hue of iridescent signals.

## Motion to conceal cues

As movement can increase conspicuousness, our understanding of adaptations to avoid detection typically assume the individual does not move, with numerous examples of colour patterns and morphological structures of immobile cryptic individuals blending remarkably with the background (Silvasti et al., 2021). However, movement does not necessarily compromise camouflage: signal attenuation at large distances means that moving objects are still harder to detect when they are similarly patterned or similar to their background (Hall et al., 2013; 2017). There are also several ways in which prey may specifically incorporate motion into their arsenal of defence strategies to compromise the cues used by their natural enemies (recently reviewed by Stevens and Ruxton, 2019 and Caro and Koneru, 2021). These mechanisms, which may operate synergistically, can be distinguished according to the two phases of natural enemy attack: the initial phase of detection of the victim, and the subsequent phase of capturing it. Thus, motion camouflage, motion masquerade and flicker-fusion effect refer to the capacity to *avoid detection*, whereas the confusion effect, motion dazzle, and deimatic or flash displays refer to the capacity to *avoid capture* following detection. Here, we simply distinguish among these mechanisms in the context of these two phases of avoiding predation, while acknowledging the ongoing debate about the terminology or complexity of these definitions (e.g. Hogan et al., 2017a and b, and Ruxton et al., 2018).

### Avoiding detection

Avoiding motion is clearly an effective way of reducing the risk of detection, and this is highlighted by the behaviour of nocturnal animals: porcupines are less mobile on moonlit nights (Mori et al., 2014), while beavers spend less time on land during brighter nights (Cabré et al., 2020). Nevertheless, other strategies have evolved to minimise cues that reveal presence, location or identity arising from a disjunct between the movement of the animal and background movement. Motion camouflage, where the nature of the movement decreases the probability of detection (Stevens and Merilaita, 2009a) can allow the moving organism to appear stationary to their targets, despite their movement (Srinivasan and Davey, 1995). For instance, dragonflies use motion camouflage to disguise their movements by imitating the trajectory of a stationary object while moving towards their targets (Mizutani et al., 2003). *Portia fimbriata* spiders in cryptic pursuit of their highly visual jumping spider (Salticidae) prey, apparently avoid detection by waving their palps and legs (Jackson, 1985). Strobing behaviour, locomotion that involves stationary pauses between bursts of very rapid movement, is a characteristic of Australian strobe ants *Opisthopsis*, and is also thought to represent a form of motion camouflage by reducing their detection by predators (Waters and McGlynn, 2018). Research using human subjects suggest that brief and rapid, random movements reduce the ability of predators to localise the prey item (Smart et al., 2020).

Animals may avoid detection as prey by mimicking an object that is uninteresting to potential predators. This deception may be enhanced by motion, defined as motion masquerade (Cuthill et al., 2019; Hall et al., 2017). Many species of stick insects sway in a side-to-side movement while otherwise remaining stationary, behaviour that is thought to reduce their conspicuousness against a background of vegetation that is moving with the wind (Bässler and Pflüger, 1979; Bian et al., 2016). Experiments by Bian et al. (2016) confirmed that swaying is maintained by random bursts of wind, and that this behaviour was quantitatively similar to that of moving plants, consistent with motion masquerade. This motion has also been interpretated as a mechanism to maintain balance (Cuthill, 2019; Kelty-Stephen, 2018), based on the incorrect premise that phasmids “perch upon a branch in windy conditions” (Kelty-Stephen, 2018). In fact, phasmids typically hang from branches to which they are firmly attached with claws. Motion masquerade may also be used by predators: swaying by praying mantids may reduce detection by prey and cannibalistic female conspecifics (Watanabe and Yano, 2013), and crypsis in predatory vine snakes may be increased by swaying in response to changes in air currents (Fleishman, 1985).

Finally, movement can reduce detection as a result of the flicker-fusion effect (Pough, 1976; Umeton et al., 2017), where the speed of movement of the object is sufficient to create a blend of colour pattern that appears very similar to its background (Endler, 1978; Stevens, 2007). For instance, experiments on mantids presented with artificial prey indicate that as speed increases, prey with contrasting patterns are more difficult to detect (Umeton et al., 2019). Depending on the receiver, this effect may be present in certain snake species: the ringed patterns of the non-venomous common water snake merge to form a single-coloured illusion when in flight (Pough, 1976), as does the zig-zag pattern of European vipers (Valkonen et al., 2020), and the patterns of coral snake mimics (Titcomb et al., 2014). However, Allen et al. (2013) found no relationship between transverse striping and speed in snakes. These contrasting results highlight the importance of interpreting signals in the context of specific receivers: the flicker-fusion effect on mammalian receivers does not necessarily apply to raptors (Valkonen et al., 2020).

### Avoiding capture

Prey animals can reduce the likelihood of capture by predators through the confusion effect, motion dazzle, and deimatic or flash displays. These mechanisms compromise information processing by predators through the presence of conspecifics and the colour patterns of the prey and motion.

Predators attempting to capture an individual from among a group of conspecific prey may be more challenged if the prey individuals are moving asynchronously (Hogan et al., 2017b; Parrish, 1993; Schradin, 2000). The reduced predation risk arising from this confusion effect is attributed to the information processing constraints of predators (Ioannou et al., 2008; Krakauer, 1995), which increase with larger numbers and/or a greater density of prey in the group (Hogan et al., 2017b; Ioannou et al., 2008; Landeau and Terborgh, 1986). For instance, stickleback attack success decreases with larger group sizes of *Daphnia magna* prey, because of increased spatial targeting error (Ioannou et al., 2008). The confusion effect relies on a high degree of similarity among individuals within the group, as ‘odd’ individuals are more readily targeted (Landeau and Terborgh, 1986). On the other hand, the confusion effect can also be enhanced by dynamic colour changes among the group-living prey (Murali et al., 2019). It would be interesting to investigate how changes in prey density, asynchronous movements of prey, and/or their dynamic colour changes have influenced the sensory capacity of the predator receivers.

Motion dazzle, where the contrasting markings of the moving prey, such as stripes or zig-zag patterns, can compromise the predator's judgement of its speed and trajectory, and thus reduces the likelihood of prey capture (Elias et al., 2019; Hämäläinen et al., 2015; Hughes et al., 2014; Kodandaramaiah et al., 2020; Murali et al., 2019; Stevens, 2007; Stevens et al., 2008; 2011; Valkonen et al., 2020). Several studies refer to this phenomenon as ‘dazzle camouflage’ (von Helversen et al., 2013; Hogan et al., 2016a,b, 2017a,b; Lingel, 2020). However, as camouflage is not necessarily the underlying mechanism reducing the risk of predation during motion dazzle (Stevens et al., 2011), we prefer ‘motion dazzle’ over ‘dazzle camouflage’ to avoid any confusion. The ‘dazzle’ aspect is induced by the high contrast markings on the prey and the success of motion dazzle may arise from reduced detection of prey by predators, regardless of the background (Hämäläinen et al., 2015). Computer-based experiments with humans reveal that the effectiveness of the motion dazzle depends upon the size of the motion dazzle patterns and the speed of the organism (Kodandaramaiah et al., 2020). Smaller organisms seem to be better protected (Kodandaramaiah et al., 2020), but the influence of the speed with which the organism moves is less clear (Kodandaramaiah et al., 2020; Stevens et al., 2008). Furthermore, high-contrast patterns may not always be effective in protecting an animal: for example, cuttlefish can adopt context-specific patterns when in motion, typically avoiding high-contrast patterns (Zylinski et al., 2009). Clearly, the effectiveness of motion dazzle depends on a range of factors, including the speed of movement and the size of the animal.

Finally, movement plays a crucial role in deimatic or startle displays, in which a camouflaged or cryptically coloured animal under imminent attack very rapidly reveals a bright colour pattern or other defence mechanism (see Umbers et al., 2017). For example, the iconic Australian frillneck lizard has a large, extensible frill around the neck that, when extended, dramatically increases the apparent size of the individual. Frillneck lizards typically extend their frills in the presence of avian predators, and in some instances the display is amplified by lunging at the predator (Perez-Martinez et al., 2020). Less dramatic versions of this behaviour may be referred to as flash displays, such as tail flashing in wading birds, or tail flagging in white-tailed deer (Caro et al., 2020). While deimatic and flash displays in the presence of a predator have the common property of facultatively revealing an otherwise concealed, conspicuous colour pattern, exactly how this behaviour reduces the probability of capture remains unclear. One possibility is that it reduces the detectability of the fleeing, less conspicuous individual (Caro et al., 2020; Loeffler-Henry et al., 2018). Perhaps the effectiveness of this defence is determined by the duration of the display, and how much time can elapse before the display ceases to have an effect on the predator's searching behaviour.

## Motion and colour pattern design

The significance of visual cues and signals is unlikely to be fully understood independently of the movement of their source or the surrounding environment. In this section, we briefly discuss some emerging predictions about the interaction of signals and the environment in the context of animal movement, which are summarised in Table 1. In particular, we explore the impact of motion on signal detail, the challenges of uncontrolled movement of the signal, and finally, the interaction between motion, signals and receivers.

**Table 1.**
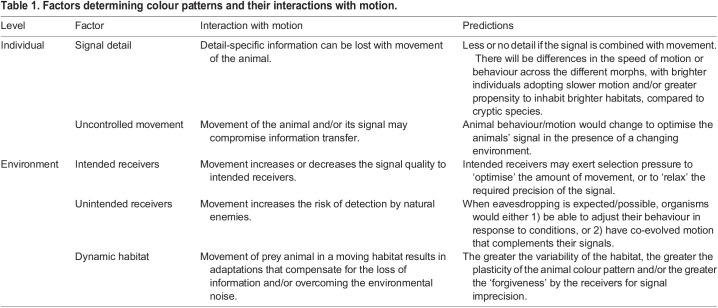
Factors determining colour patterns and their interactions with motion.

### Signal detail

Many visual signals include detailed patterns arising from a combination of contrasting colours and colour characteristics, and the information conveyed by such signals may be specified by the detail of these colour pattens (Table 1). Small deviations from even simple colour patterns can have life or death consequences for many organisms, ranging from chemically defended beetle larvae (Tan et al., 2016) to the eggs of brood parasite cuckoos (Honza and Cherry, 2017). The degree of mimicry in the eggs of cuckoo brood parasites is truly remarkable, and perhaps necessary because every cuckoo egg ‘encounters’ the host parent, who can very carefully scrutinise the details of the patterns of the stationary eggs. This degree of scrutiny may not be possible for *Malurus* fairy-wrens, whose domed nests create a dim interior that may make it difficult to discriminate their eggs from those of bronze cuckoo parasites. Discriminating between moving chicks is likely to be even more challenging, and may contribute to the evolution of a different sensory modality: adults use begging calls, rather than visual traits to discriminate between host and bronze cuckoos nestlings (Langmore et al., 2003). Garcia et al. (2020), drawing on insights from psychophysics, emphasise the importance of considering observer perception when considering the biological significance of differences in the colour patterns of visual mimetic systems, and further insights seem likely by incorporating the movement of both the signal and observer within this paradigm.

The details in the colour pattern that acts as a signal may depend upon the speed of the animals' movement (Table 1). Very rapid movement may blur the detail of the colour pattern, thereby concealing the information the signal is supposed to convey. This may be especially apparent under sexual selection, which can favour the evolution of elaborate visual signals in one sex, typically males, that provide information about their relative suitability as mates to discerning females (Anderssen, 1994; Hare and Simmons, 2019; Wiens and Tuschhoff, 2020). In some species, these colour patterns may be remarkably detailed, such as those of *Maratus* peacock spiders (Stavenga et al., 2016), or the detailed patterned plumage of estrildid finches (Soma and Garamszegi, 2018). In other species, the colour patterns may be far less detailed, such as the gorgeous feather colours of some birds of paradise that are highlighted by elaborate courtship movements (e.g. Ligon et al., 2018). If rapid movement blurs the detail of the colour pattern, then the information the signal is supposed to convey may be lost. Accordingly, we predict that the degree of detail of animal colour patterns, or their function as revealing information about the courting male, is associated with the speed or nature of movement that occurs during courtship displays. Courtship that involves rapid movement may constrain the level of colour pattern detail or, conversely, the courtship displays of males of species with detailed colour patterns may involve less rapid movement. For example, male fairy-wrens hold flower petals in their bill while courting the female (e.g. Karubian and Alvarado, 2003), and the frequent movement of the male during courtship (Mulder, 1997) suggests that the relevant information is conveyed by the simple possession of the petal, rather than any details of its shape, size or colour.

The predicted association between signaller movement and signal detail can be generalised to other signalling contexts (Table 1). The detail of a colour pattern necessary for prey to convey information about their palatability (e.g. Maan and Cummings, 2012) or ease of capture (e.g. Páez et al., 2021) may also vary according to the movement of the signaller. Species-level comparisons across several different taxonomic groups reveal differences in colour patterns according to mobility: among lizards, species with striped colour patterns are more mobile than those with cryptic patterns (Halperin et al., 2017); small, fast-moving snakes that hunt actively tend to have longitudinal stripes, while large, slow snakes that ambush hunt have blotched patterns (Allen et al., 2013); longitudinal bands occur more frequently on the eye-lines of faster moving, slender fish species, and vertical bands occurred more frequently on sharply turning, deep-bodied fishes (Barlow, 1972). While these inter-specific associations suggest relationships between movement and colour pattern detail, it is unclear whether these colour patterns act as conspicuous signals or concealing cues. Variation within species may provide clearer insights. For example, there is remarkable between-population variation in the colour patterns of the poison arrow frog *Dendrobates pumilio*, which is correlated with chemical defence (Maan and Cummings, 2012). The mobility of the frog may also contribute to this variation: perhaps frogs with more detailed colour patterns are also generally less mobile.

### Uncontrolled signal movement

Signallers may have to contend with uncontrolled movement of their signal, and thus the information it conveys (Table 1). For example, male greater bowerbirds create a visual illusion for females viewing decorations located in the ‘court’ beside the bower, by placing different sized decorations at different distances from the bower, and the quality of this illusion determines male mating success (Kelley and Endler, 2012). However, these decorations may be displaced by the elements or by the movement of other animals across the court, thus altering the quality of the illusion, and so males must monitor the court and, if necessary, adjust the arrangement of any disturbed decorations (Kelley and Endler, 2012).

For some species, adjustments to compensate for uncontrolled movement may not always be possible. Many species of orb-weaving spiders incorporate decorations, comprising conspicuous silks or prey debris, around the centre of their orb-webs (Herberstein et al., 2000; Tan et al., 2010; Théry and Casas, 2009; Walter and Elgar, 2012). The presence of these decorations is puzzling because the effectiveness of an orb-web would seem to depend upon being inconspicuous, which might be compromised by these decorations (Walter and Elgar, 2012). Empirical studies that address the function of these decoration signals provide inconsistent evidence, encouraging a lively debate (Herberstein et al., 2000; Tan et al., 2010; Théry and Casas, 2009; Walter and Elgar, 2012). Perhaps these differences arise from variation in the degree of uncontrolled movement of the decoration and thus their efficacy as a signal (Table 1). Orb-webs are remarkably flexible and may move in response to quite light air currents, causing movement of the decoration signal. There is considerable variation in the conspicuousness of these decorations (and the resident spiders, see Peng et al., 2020) to receivers with different visual acuities, as inferred from stationary images (Caves et al., 2018), and this conspicuousness may vary even more as the wind moves both spiders and background vegetation. How uncontrolled movement could affect the efficacy of the decoration signal remains unknown and deserves further investigation.

### Receivers

The degree to which patterns of motion (including speed, orientation and repeatability) influence the effectiveness of the colour pattern depends in part on the receiver: natural enemies will exert strong selection favouring motion patterns that optimise the effectiveness of either the signal (e.g. revealing chemical defence) or the concealing colour pattern. However, receivers in other signalling contexts may be more ‘forgiving’ and still recognise the information conveyed by colour patterns that are slightly distorted by any movement (Table 1). While technical advances allow us to ask questions about the neurological processes associated with detecting visual signals (e.g. Drewniak et al., 2020; Ito et al., 2021; Silvasti et al., 2021), less attention has focussed on the impact of signal imprecision, especially that introduced by movement, and how it is accommodated before the ‘signal’ fails to convey information. Mimetic systems may be especially useful models for investigating the impact of motion on colour patterns that provide protection from natural enemies. For example, the octopus, *Macrotritopus defilippi*, mimics both the swimming behaviour and colouration of its flounder model (Hanlon et al., 2010), indicating that mimicry of the colour pattern alone provides insufficient defence against predators. Likewise, the remarkable visual resemblance of ant-mimicking salticid spiders to their ant models may also be insufficient: some species also move their first pair of legs to mimic the antennal movement of ants (Ceccarelli, 2008).

Visual signalling that is vulnerable to eavesdroppers may be helpful systems for investigating the links between movement, visual display and receiver visual acuity because the latter may differ between intended and unintended receivers. Eavesdroppers are unintended receivers of a signal (McGregor et al., 1993; Brandley et al., 2013), and movement that increases the conspicuousness of the signal for the intended receiver may or may not act similarly for eavesdroppers (Table 1). First, the frequency of movement required to alert the intended receiver may not be the same as that required to alert the eavesdropper. Field experiments with robotic lizard signallers suggest that the risk of attack is not substantially increased when the dew-lap display, which is exposed by movement, is relatively infrequent (Ord et al., 2021). Second, the display may convey different information to the intended receiver and eavesdropper. Male fireflies produce flashes of bioluminescence while flying, and the patterns of light production provide information to conspecific females about the nature or attributes of the male, but his location to predatory eavesdroppers (Stanger-Hall and Lloyd, 2015). Intriguingly, these predatory eavesdroppers can include other species of fireflies: female *Photorius* fireflies are specialist predators of *Photinus* fireflies, locating their prey by eavesdropping on the visual signal (e.g. Lloyd, 1973; Lewis and Cratsley, 2007). Phylogenetic comparative analyses indicate evolutionary associations between flashing patterns and movement (Stanger-Hall and Lloyd, 2015), and between flashing patterns and sensory (eye) morphology (Stanger-Hall et al., 2018). Perhaps these patterns also vary with the risk of eavesdropping. Intriguingly, firefly flashes may function beyond mate attraction: there is some evidence that firefly flashes represent an aposematic signal to spiders (Long et al., 2012) and bats (Moosman et al., 2009). Perhaps the timing of these flashes, and the movement of males and females, align with the perceptual capacity of intended conspecific and heterospecific receivers, and other eavesdroppers.

## A dynamic environment

Some forty years ago, John Endler (1978) highlighted the importance of the background environment for shaping both conspicuous signals and concealing cues – insights that continue to have a profound impact on our understanding of organismal colour patterns and visual ecology more generally. Nevertheless, the environment is rarely static: animals move, which means their background structure may vary over time; and the background itself may move if it comprises flexible, non-rigid structures such as vegetation. Despite the increasing interest in investigating how the movement of both animals and their background has a concealing function (recently reviewed by Cuthill et al., 2019), there has been less focus on how movement affects visual signals. Examples of the importance of these interactions abound – more complex habitats can function to conceal signals: lizards adjust their displays with varying wind conditions to increase signal efficacy (Bian et al., 2019; Ord et al., 2007; Ord and Stamps, 2008; Ramos and Peters, 2017), while habitat structure drives the evolution of aerial sexual displays in birds (Menezes and Santos, 2020). In aquatic habitats, where the light intensity fluctuates and is polarised, visual signals and cues are affected by both the movement of water and the movement of the animal (Cuthill et al., 2019). For example, dappled light can mask the movement of prey and thus disrupt detection (Matchette et al., 2019), while selection on reef fish mimics to resemble their model varies with the depth of water and thus light environment (Cheney and Marshall, 2009). Clearly, investigations of the significance of visual signals and cues must incorporate a moving source against a dynamic, moving background. The movement of both animals and their background thus has implications for both the conspicuousness of visual signals and the effectiveness with which colour patterns conceal cues (Table 1). While it is widely understood that the impact of motion on revealing cues are the mirror image of those for concealing signals, our review highlights how motion can make signals more conspicuous and cues less conspicuous.
